# Online Tobacco Advertising and Current Chew, Dip, Snuff and Snus Use among Youth and Young Adults, 2018–2019

**DOI:** 10.3390/ijerph19084786

**Published:** 2022-04-14

**Authors:** Megan C. Diaz, Elexis C. Kierstead, Domonique Edwards, Yoonsang Kim, Shyanika W. Rose, Sherry Emery, Bushraa Khatib, Michael Liu, Ganna Kostygina

**Affiliations:** 1 Schroeder Institute, Truth Initiative, Washington, DC 20001, USA; lkierstead@truthinitiative.org (E.C.K.); domonique.dae@gmail.com (D.E.); bkhatib@truthinitiative.org (B.K.); mliu@truthinitiative.org (M.L.); 2 NORC at the University of Chicago, Chicago, IL 60637, USA; kim-yoonsang@norc.org (Y.K.); emery-sherry@norc.org (S.E.); kostygina-anna@norc.org (G.K.); 3 College of Medicine, Department of Behavioral Science, Center for Health Equity Transformation, University of Kentucky, Lexington, KY 40506, USA; s.rose@uky.edu

**Keywords:** smokeless tobacco, tobacco advertising, social media, policy

## Abstract

Objective: To understand the relationship between exposure to online tobacco advertising and current smokeless tobacco use in the context of tobacco control policies. Methods: Three waves of a national probability-based sample of (*n* = 15,985) youth and young adults were used. Analysis consisted of GEE logistic models controlling for social media use, demographics, tobacco use, average price of smokeless tobacco inclusive of taxes, smoke-free indoor air laws (SFIA) and state tobacco control expenditures. Results: Frequent exposure to tobacco advertising on social media is associated with greater odds of current smokeless use (aOR: 2.05, 95% CI: 1.62, 2.60). Higher prices and SFIA coverage were associated with reduced current smokeless use when examined separately from other tobacco policy variables (aOR: 0.79, CI: 0.73, 0.85; aOR: 0.44, CI: 0.28, 0.70). Conclusions: Greater exposure to tobacco advertising online is associated with greater odds of smokeless use among surveyed youth and young adults. This effect of social media marketing exposure on smokeless use outweighs the mitigating impact of existing tobacco control policies. The findings underscore the need for strong advertising regulation of evolving tobacco products, including smokeless products, on social media and surveillance of digital marketing tactics to young people.

## 1. Introduction

Despite declining cigarette use and the rapid rise of e-cigarette use among young people, youth use rates of smokeless tobacco products, specifically chew, dip, snuff and snus, have remained stable in the United States (U.S.) in the past decade. As of 2020, 5% of 8th, 10th and 12th graders used smokeless tobacco products [1]. Smokeless tobacco use is highest among White males living in rural areas, and the product has been historically marketed to cigarette users as a non-combustible tobacco alternative for use where combustible tobacco is restricted but is now being leveraged to youth who may no longer be attracted to combustible cigarettes [2].

Tobacco companies are increasingly marketing their products on social media platforms most popular with young customers [3]. Their extensive reach among youth [4] make social media platforms a powerful tool for communicating social norms and influencing risk behaviors among youth [5,6]. A 2015 study found that 52.5% of youth reported exposure to tobacco imagery via social media in the past month and a 2019 study found that 21% of youth reported actively engaging with at least one form of online tobacco marketing [7]. Youth who had engaged with online tobacco marketing were more likely to initiate tobacco, use tobacco more frequently, use multiple tobacco products and were less likely to later quit [8]. Additionally, youth who reported two or more forms of online tobacco marketing engagement were more likely to use smokeless tobacco products a year later [9].

Research shows that engaging with promotional messages on social media is linked to increased tobacco product susceptibility among never users, emphasizing the impact of tobacco advertising via online platforms [7]. These promotional messages can include content created and distributed by the company, paid influencers and product users. Exposure to product placements in social media feeds may also directly influence knowledge, attitudes and beliefs or could strengthen the relationship between such beliefs and tobacco-related behaviors [7,10,11]. With the recent rise of social media use, tobacco companies are leveraging social media that is popular among youth to cultivate a younger customer base addicted to alternative tobacco products [3,12].

Despite its pervasive impact, tobacco advertising on social media is under-regulated, creating space to present products like smokeless tobacco to young audiences. Social media was not present when either the 1998 Smokeless Tobacco Master Settlement Agreement or the 2009 Tobacco Control Act were passed, restricting tobacco advertising to outdoor and transit advertising, or distribution of apparel or merchandise [13,14,15,16]. Although many social media platforms ban paid tobacco advertising, to varying degrees of enforcement, product promotions circulated by branded social media accounts and paid product influencers are less regulated [11,17], prompting tobacco companies to take advantage of patchwork regulation to raise brand visibility and engagement [18,19].

Despite evidence of the power of social media tobacco marketing, more research is needed to understand the direct effect of exposure on tobacco use behaviors—specifically on smokeless tobacco use. This study aimed to explore the relationship between self-reported digital tobacco marketing exposure and current smokeless tobacco use, using data from a nationally representative, longitudinal sample of U.S. youth and young adults. We also aimed to understand to what extent tobacco control policies including prices, smoke-free indoor air laws (SFIA) and tobacco control expenditures modify the relationship between self-reported digital tobacco marketing exposure and smokeless tobacco use.

## 2. Materials and Methods

### 2.1. Sample

Data were obtained from the Truth Longitudinal Cohort (TLC), a national probability-based youth and young adult cohort (ages 15–21 at recruitment) established to evaluate the tobacco prevention media campaign, “truth^®^”. Participants were primarily recruited via address-based sampling from a customized panel from GfK KnowledgePanel, with follow-up online surveys every six months to one year. In most survey waves, new participants were recruited, spanning anywhere from 400–1300 individuals, to reduce bias due to attrition and help cross-sectional representation of the sample. Wave 7 was fielded from 15 February to 29 May 2018; Wave 8 was fielded from 10 February to 20 May 2019; and Wave 9 was fielded from 26 August to 16 December 2019. In Wave 7, questions were added to examine awareness of tobacco advertising. We included all participants at Wave 7 (*n* = 14,377), Wave 8 (*n* = 12,113) and Wave 9 (*n* = 10,902) to maximize our number of observations. Sampling methods are described elsewhere [20,21,22]. Survey retention rates were 72.7% for Wave 7, 67.5% for Wave 8 and 66.4% for Wave 9.

### 2.2. Measures

Respondents who had ever used chewing tobacco, dip, snuff and snus were asked on how many of the past 30 days they used these products. Participants were considered current users if they reported use on 1–30 days and non-current users if they responded with “0 days” or had never used smokeless tobacco. Smokeless tobacco use served as the primary outcome.

Tobacco advertising exposure on websites and social media was the primary independent variable. For Waves 7 and 8 participants were asked “How often do you remember seeing or hearing about tobacco advertising or promotions on websites or social media in the past 30 days?” For Wave 9, tobacco advertising exposure in the past 30 days on websites and social media was evaluated using two separate questions (for websites and social media, respectively). To account for this data difference, we used the maximum response for either question to match the response categories used for Waves 7 and 8. Response options were categorized: never, sometimes and often/very often.

In our models we controlled for a series of independent variables suggested by the literature to affect smokeless tobacco use. We started by controlling for time spent on social media to account for the possibility that the intensity of social media use may confound the relationship between an individual’s tobacco advertising exposure, their memory of this exposure and smokeless product use. Participants were asked how much time they spent on social media on an average day. Response options included: none, less than 1 h, 1 to less than 3 h and 3 h or more.

We also controlled for: respondent age at each wave, gender (female, male), race/ethnicity (any non-White race/ethnicity combined, non-Hispanic White), highest educational attainment of either parent (less than high school education, high school graduate, some college or associates degree, college graduate or more) and non-metropolitan residence determined if an individual’s home address was located in a non-metropolitan statistical area as classified by the United States Census Bureau (non-metropolitan, metropolitan).

To control for the respondent’s tobacco environment we included own poly-tobacco use, any household tobacco use and peer cigarette smoking. Own poly-tobacco use was defined as “yes” if participants used cigarettes, large cigars or cigarillos and/or electronic cigarettes in the past 30 days, and “no” if they had not. Household tobacco use was defined as “yes” if those living in the same household as the survey participant used cigarettes, large cigars or cigarillos, hookah or e-cigarettes, and “no” if no household members used any of the previously listed tobacco products. To assess peer cigarette smoking, participants were asked how many of their four closest friends smoked cigarettes; response options were treated categorically in the model.

As sensation-seeking tendencies are known to affect tobacco use [23], we included a validated sensation-seeking index constructed from eight survey items with a five-point Likert scale (strongly disagree to strongly agree) [24]. A description of this measure is published elsewhere [24]. The mean score of the scale items was calculated and treated as a continuous measure with a range of (1–5); the measure was assessed upon entry to the cohort and is time-invariant in our models.

In addition to demographic variables, it has been proven that tobacco control policies impact smokeless tobacco use [25]. Thus, we controlled for the policy environment by including three state-level measures as time-varying covariates: (a) price of smokeless tobacco, (b) share of the population covered by indoor smoking restrictions, and (c) state tobacco control expenditures. Tobacco control expenditure data were aggregated at the calendar year. Data from 2018 were assigned to Wave 7, and data from 2019 were assigned to Waves 8 and 9. First, we used Nielsen retail scanner data to estimate the price of one ounce of smokeless tobacco in each state. We summed the total dollar sales and total ounces sold for the ten most popular brands based on total dollar sales. We then calculated the sales weighted average price of one ounce of smokeless tobacco in each state where the participant resided for the four-week period in which they submitted the survey. State-level sales data were available for the 48 continental states. We adjusted for inflation (2019 dollars) using the United States Bureau of Labor Statistics Consumer Price Index [26]. This price measure is inclusive of all taxes levied.

Secondly, we summed to the state level the share of the population in each county covered by smoke-free indoor air laws (SFIAs) for private workplaces, restaurants and bars. These data have been used elsewhere [27], and weights SFIAs applying to bars, restaurants and private workplaces equally, while partial SFIAs are weighted by half.

Thirdly, we constructed a per capita measure of state tobacco control expenditures adjusted for inflation (2019 dollars) and based on the American Lung Association’s annual State of Tobacco Control report. We assigned aggregated fiscal year data from 2018 to Wave 7, data from fiscal year 2019 to Wave 8 and data from fiscal year 2020 to Wave 9. This report includes spending for each state from tobacco excise tax revenues earmarked for tobacco control, Master Settlement Agreement funds, individual state settlements with the tobacco industry earmarked for tobacco control, other state funds appropriated for tobacco control programs, and Federal funding to states allocated for tobacco control [28,29]. We used the United States Census decennial estimates to calculate per capita figures.

### 2.3. Analysis

Characteristics of respondents were summarized for each wave. Frequency and percentage were reported for categorical variables; mean and standard deviation were reported for continuous variables. Characteristics included tobacco advertising exposure, current smokeless use, social media use, demographic and psychosocial characteristics, and tobacco policy measures.

We used a generalized estimating equation (GEE) for logistic regressions to evaluate the relationship between self-reported tobacco advertising exposure on websites and social media and current smokeless use. GEE regression models account for lack of independent outcomes across waves within participants [30]. We used an exchangeable correlation structure in our models to account for correlated errors across waves within participants, and calculated robust standard errors using the method developed by Huber [31]. We did not account for complex survey design because there is no readily available software to correctly calculate standard errors while simultaneously accounting for the survey design and correlated errors nested within participants.

We estimated five alternative models. Model 1 controlled for social media exposure, age, gender, parental education, race, sensation-seeking tendencies, residence in a non-metropolitan statistical area, own poly-tobacco use, household tobacco use and peer cigarette smoking. To understand to what extent tobacco control policies modify the relationship between self-reported tobacco marketing exposure and smokeless tobacco use, Models 2 through 4 included all aforementioned variables and each of the state-level policy measures individually. These three measures were moderately correlated from 0.28–0.47 and thus included in separate models to minimize collinearity. However, omitting these variables could result in biased estimates of the effect of tobacco advertising exposure on current smokeless tobacco use; therefore, Model 5 included all covariates. We present alternative models in this manner to exemplify the trade-off between multicollinearity and potential omitted variable bias. We present four additional models in a supplemental table. Model 6, our simplest model, only includes our primary outcome, exposure to tobacco advertising and wave fixed effects. We subsequently include social media use (Model 7), and our demographic and psychosocial characteristics (Model 8). We also include a model with all covariates and state fixed effects (Model 9). All analyses were conducted using Stata version 15.1.

## 3. Results

### 3.1. Sample

There were 15,985 unique respondents across the three waves, 23% (*n* = 3675) had data for one wave only, 20% (*n* = 3213) had data for two waves and 57% (*n* = 9097) had data in all three waves.

Across the three waves, respondents were on average 23 years old (spanning in age from 15–36), less than half were male, 64–65% were non-Hispanic White and 58–59% of parents completed some college education or more (Table 1).

Any household tobacco use changed over time, decreasing from 39% in Wave 7 to 20% in Wave 9, while own poly-tobacco use and peer cigarette use remained steady. For 67–70% of participants, none of their four closest peers smoked cigarettes, while 18–19% reported that one of their friends smoked, about 8% reported two of their friends smoked, about 3% reported three of their friends smoked and 2–3% reported four of their friends smoked (Table 1).

### 3.2. Smokeless Tobacco Use, Tobacco Advertising Exposure and Social Media Use

Across the survey waves, about 2% of the respondents used smokeless tobacco products in the past 30 days. Regarding their exposure to tobacco advertising on websites and social media, 27–34% reported they were sometimes exposed and 6–7% said they were often or very often exposed. About 32–37% of the respondents reported being on social media not at all or <1 h a day, 43–44% reported spending 1–3 h a day and 20–24% reported spending 3+ h a day (Table 1).

### 3.3. Policy Environment

Across the waves 83–84% of participants were covered by SFIAs (Table 1). Real price of smokeless tobacco products was USD 4.57–4.89 per ounce, and state tobacco control expenditure was on average USD 2.25 per capita (Table 2).

### 3.4. Modeling

Self-reported exposure to tobacco advertising on websites and social media was significantly associated with current smokeless tobacco use across all five models with an adjusted odds ratio (aOR) of 2.05–2.07 (Table 3). Additionally, compared to individuals who use social media 3+ h a day, those who reported no daily social media use had significantly lower odds of current smokeless tobacco use (aOR: 0.65–0.66) (Table 3). The stability of these effects across each model strongly indicates that the effects are independent of other variables and not the result of confounding. Without including our covariates, the adjusted odds ratio is 2.47 (Supplementary Table S1, Model 6).

Respondents living in a non-metropolitan statistical area (aOR: 1.90–2.09) had higher odds of current smokeless tobacco use. Conversely, respondents who are non-Hispanic White (aOR: 1.73–1.79), male (aOR: 5.51–5.56), poly-tobacco users (aOR: 2.98–3.00), living with a tobacco user (aOR: 1.27) and have higher sensation-seeking tendencies (aOR: 1.41–1.43) had greater odds of current smokeless tobacco use. Further, respondents with peers who smoke cigarettes had greater odds of smokeless use, and the odds of current smokeless use increased with the number of peers who smoked cigarettes; for those with two or more peers smoking cigarettes, the odds were almost two and a half times that of those with no friends who smoked cigarettes.

The three state-level policy measures showed varying effects across the models. When price of smokeless tobacco was included as the sole policy variable (Model 2) and amongst the other policy variables (Model 5), the odds of current smokeless tobacco use were 31% lower with one dollar increase in price. The odds were 56% lower for those residing in a state with SFIA policies, compared to those without (Model 3). State tobacco control expenditure, however, was not significantly associated with current smokeless tobacco use (Model 4). When all policy variables were included in the model (Model 5), the effects of SFIA policies and state tobacco control expenditures were insignificant. The lack of significance of these variables is possibly due to multicollinearity. To test this, we calculated mean variance inflation factors (VIFs). Mean VIFs for Models 1–5 was 1.2, suggesting that multicollinearity is not an issue. Wave fixed effects were also included in all models; there was no significant change in the current smokeless tobacco use over waves. We also examined a model where we included state fixed effects; however, we present these results in our supplemental table due to high multicollinearity. The results from this model are consistent with all five models presented.

## 4. Discussion

Youth and young adults in this study often or very often exposed to tobacco advertising on websites and social media had more than double the odds of being current smokeless users compared to those who were not exposed. Although not statistically significant, the odds were 3% greater for those who were sometimes exposed to tobacco advertising than those who were not exposed. Trends persisted when accounting for social media use, demographics, psychosocial and tobacco use characteristics, as well as each policy variable of interest individually. These findings are consistent with prior literature, as increased exposure to online tobacco advertising is associated with increased initiation and use of tobacco and nicotine products among youth [8,32,33,34,35,36]. Also consistent with the literature, White male participants living in rural areas and those using multiple tobacco products had greater odds of smokeless tobacco use [2,8,37]. While national studies find that smokeless use is highest in the 25–44 age group [38], we focused on youth and young adults as an at-risk population for establishing patterns of smokeless tobacco use and susceptibility to social media advertising. In our study, marketing exposure and smokeless tobacco use were measured within each wave over time allowing for the possibility that those using smokeless tobacco may seek out marketing. However, we believe this may not be the case, as prior research on other forms of marketing suggests that marketing exposure is cumulative over time. As an example, youth receiving tobacco coupons at baseline and one year later were more likely to use smokeless at follow-up and these effects were stronger for those who were not using at baseline [9].

Our findings suggest that the amount of time spent on social media matters. Youth and young adults in this study who spent three or more hours per day on social media had 51% greater odds of smokeless tobacco use than those who did not spend any time on social media.

Smokeless tobacco use among youth and young adults remains a growing concern. As youth lean away from cigarettes, some are using a diverse array of new nicotine products [12,39] such as Zyn, a novel smokeless product touted as “tobacco-free” [40,41]. Zyn sales increased by 470% in the first six months of 2020 [42]. Products like Zyn should be monitored, specifically among youth and young adults, as their emergence indicates an industry interest to expand the market for alternative non-combustible products or as an alternative to quitting [43].

In addition to expanding their product market, tobacco companies are increasingly dedicating resources to promote smokeless products on smartphone-optimized websites, apps and social media [34,44]. This is concerning given that youth and young adults use social media at disproportionately high rates [4], as well as the pre-existing findings that tobacco advertising on social media encourages product use [18,19], and the direct relationship between tobacco promotion and smokeless use observed in this study.

Our results also confirm that tobacco control policies curb youth and young adult smokeless use. Higher prices and SFIA laws were independently associated with lower odds of smokeless tobacco use. Prior research has shown the relationship between smokeless price and product use and has identified that SFIA laws are indicative of a stronger tobacco regulatory environment, decreasing the likelihood of tobacco use [45,46]. With the adjustment of tobacco control policies in the models, exposure to tobacco advertising still had a significant effect on smokeless use, emphasizing the significant power of tobacco advertising on social media. These findings underscore the need for strong policies banning online tobacco marketing to youth and young adult populations—akin to historic bans on cigarette marketing—to meaningfully reverse trends in smokeless tobacco product use.

## 5. Limitations

Although this study has many strengths, it is not without limitations. The data available with our measures of interest were limited to a time span of a year and a half, restricting our ability to observe the relationship over time. Second, the survey design involved multistage sampling and over-sampling of sub-populations, normally accounted for using sample weights. We did not account for sampling weights in the GEE logistic models as there is no readily available statistical software to correctly estimate the standard errors of odds ratios from logistic regression while simultaneously accounting for complex survey design and correlated errors nested within respondents. To reduce the bias, we controlled for respondents’ demographic characteristics that were related with survey sampling and weighting and applied robust standard errors [31]. We acknowledge that the magnitudes of odds ratios may not be generalizable to the U.S. youth and young adult population; however, the positive relationship found between exposure to tobacco advertising and smokeless tobacco use still holds. Additionally, our study used self-reported measures of tobacco advertising exposure on websites and social media, which could be subject to recall bias. The validity of this measure requires respondents to see an advertisement, recognize it as a tobacco ad, encode the image in memory and then retrieve the image from memory when prompted by a survey question [47]. Further, the self-reported exposure measure may suffer from endogeneity: respondents who are or are interested in using smokeless tobacco will have greater opportunities to be exposed to online tobacco advertisements due to their online search behavior [48]. Therefore, it would be ideal to use self-reported recall measures in conjunction with exogenous and objective assessment of the level of exposure in a given geographic region and time period. Lastly, our survey question pertaining to tobacco advertising exposure was not specific to smokeless tobacco products.

## 6. Conclusions

The strong relationship between exposure to online tobacco marketing and smokeless tobacco use emphasizes the importance of social media and websites as a key promotional arena for tobacco companies, particularly when considering the expansion of the alternative, non-combustible tobacco product market in recent years. Findings underscore the need for stronger regulation of tobacco product marketing on digital platforms to curtail the impact of these promotional activities on young people.

## Figures and Tables

**Table 1:** Categorical sample characteristics across Waves 7, 8 and 9 of the national Truth Longitudinal Cohort.

	Wave 7 February–May 2018 Response Rate: 72.7%		Wave 8 February–May 2019 Response Rate: 67.5%		Wave 9 September–December 2019 Response Rate: 66.4%		Wave 7–9	
Current Smokeless Use	*n*	*%*	*n*	*%*	*n*	*%*	*n*	*%*
Yes	281	2.0	225	1.9	211	1.9	717	1.9
No	14,084	98.0	11,887	98.1	10,691	98.1	36,662	98.1
Tobacco Advertising Exposure	*n*	*%*	*n*	*%*	*n*	*%*	*n*	*%*
Never	9399	66.0	8099	67.4	6415	59.0	23,913	64.4
Sometimes	3890	27.3	3253	27.1	3669	33.7	10,812	29.1
Often, Very often	956	6.7	657	5.5	796	7.3	2409	6.5
Social Media Use	*n*	*%*	*n*	*%*	*n*	*%*	*n*	*%*
None	970	6.8	878	7.3	772	7.1	2620	7.0
Less than 1 h	4325	30.3	3004	24.9	2749	25.3	10,078	27.1
1 to 3 h	6150	43.1	5271	43.6	4774	43.9	16,195	43.5
3 or more h	2840	19.9	2935	24.3	2585	23.8	8360	22.4
Gender	*n*	*%*	*n*	*%*	*n*	*%*	*n*	*%*
Male	6233	43.4	5208	43.0	4636	42.5	16,077	43.0
Female	8144	56.7	6905	57.0	6266	57.5	21,315	57.0
Parental Education	*n*	*%*	*n*	*%*	*n*	*%*	*n*	*%*
LT high school	673	4.8	514	4.3	468	4.4	1655	4.5
High school graduate	1860	13.2	1504	12.7	1380	12.9	4744	12.9
Some college/AA degree	3414	24.2	2822	23.7	2529	23.6	8765	23.9
College graduate or more	8142	57.8	7051	59.3	6347	59.2	21,540	58.7
Race/Ethnicity	*n*	*%*	*n*	*%*	*n*	*%*	*n*	*%*
Non-Hispanic White	9220	64.2	7860	64.9	7011	64.3	24,091	64.4
Any other race	5152	35.9	4250	35.1	3891	35.7	13,273	35.6
Metropolitan Residence	*n*	*%*	*n*	*%*	*n*	*%*	*n*	*%*
Metropolitan	10,987	88.8	10,797	89.1	9738	89.3	31,522	89.1
Non-metropolitan	1385	11.2	1316	10.9	1164	10.7	3865	10.9
Own Poly-Tobacco Use	*n*	*%*	*n*	*%*	*n*	*%*	*n*	*%*
None	11,455	79.7	9372	77.4	8614	79.0	29,441	78.8
Any (cigarette, cigar, ENDS)	2922	20.3	2737	22.6	2285	21.0	7944	21.2
Household Tobacco Use	*n*	*%*	*n*	*%*	*n*	*%*	*n*	*%*
None	8722	61.1	9424	78.7	8688	79.8	26,834	72.3
Any	5549	38.9	2557	21.3	2199	20.2	10,305	27.8
Peer Smoking	*n*	*%*	*n*	*%*	*n*	*%*	*n*	*%*
None	9523	67.0	8357	69.9	7548	69.4	25,428	68.6
1	2701	19.0	2156	18.0	2019	18.6	6876	18.6
2	1179	8.3	923	7.7	817	7.5	2919	7.9
3	457	3.2	314	2.6	300	2.8	1071	2.9
4	356	2.5	213	1.8	188	1.7	757	2.0

**Table 2:** Continuous sample characteristics across Waves 7, 8 and 9 of the national Truth Longitudinal Cohort.

	Wave 7 February–May 2018 Response Rate: 72.7%		Wave 8 February–May 2019 Response Rate: 67.5%		Wave 9 September–December 2019 Response Rate: 66.4%		Wave 7–9	
	Mean	SD	Mean	SD	Mean	SD	Mean	SD
Age	22.2	4.2	23.0	4.0	23.6	4.1	22.9	4.1
Sensation Seeking	2.9	0.8	2.9	0.8	2.9	0.8	2.9	0.8
Real Weighted Avg Price of Smokeless Tobacco (USD/one oz) *	$4.57	1.44	$4.75	1.51	$4.89	1.56	$4.72	1.5
Smoke-Free Indoor Air Laws (% population)	83.3%	20.7	83.5%	20.5	83.9%	20.5	83.5%	20.6
Real State Tobacco Control Expenditure (USD/capita) *	$2.41	2.52	$2.15	$1.93	$2.16	1.94	$2.25	2.18

* Adjusted for inflation to 2019 dollars.

**Table 3:** Current smokeless tobacco use modeled against tobacco advertising expenditure, social media use, sociodemographic characteristics and policy variables using Waves 7,8 and 9 (2017–2019) of the national Truth Longitudinal Cohort Study.

	Model 1	Model 2	Model 3	Model 4	Model 5
	OR	OR	OR	OR	OR
	95% CI	95% CI	95% CI	95% CI	95% CI
Tobacco Advertising Exposure (REF: Never)					
Sometimes	1.03	1.03	1.03	1.03	1.03
	(0.87, 1.23)	(0.87, 1.23)	(0.87, 1.22)	(0.87, 1.23)	(0.87, 1.23)
Often, Very often	2.07 ***	2.05 ***	2.07 ***	2.06 ***	2.05 ***
	(1.63, 2.62)	(1.61, 2.60)	(1.63, 2.63)	(1.63, 2.62)	(1.62, 2.60)
Social Media Use (REF: 3 or more h)					
None	0.65 *	0.65 *	0.66 *	0.65 *	0.66 *
	(0.44, 0.97)	(0.44, 0.98)	(0.44, 0.98)	(0.44, 0.98)	(0.44, 0.98)
Less than 1 h	0.96	0.95	0.96	0.96	0.95
	(0.77, 1.19)	(0.76, 1.19)	(0.77, 1.21)	(0.77, 1.20)	(0.76, 1.19)
1 to 3 h	0.99	0.98	1	0.99	0.98
	(0.81, 1.19)	(0.81, 1.19)	(0.82, 1.21)	(0.82, 1.20)	(0.81, 1.19)
Age	1.02	1.02	1.02	1.02	1.02
	(0.99, 1.05)	(0.99, 1.05)	(0.99, 1.05)	(0.99, 1.05)	(0.99, 1.05)
Gender (REF: Female)					
Male	5.54 ***	5.52 ***	5.55 ***	5.56 ***	5.51 ***
	(4.31, 7.14)	(4.28, 7.12)	(4.31, 7.15)	(4.31, 7.16)	(4.28, 7.11)
Parental Education (REF: College Graduate or More)					
Less than high school	1.25	1.31	1.26	1.25	1.31
	(0.75, 2.10)	(0.78, 2.20)	(0.75, 2.11)	(0.75, 2.10)	(0.78, 2.20)
High school graduate	1.17	1.13	1.15	1.16	1.13
	(0.85, 1.60)	(0.82, 1.55)	(0.84, 1.57)	(0.85, 1.59)	(0.82, 1.55)
Some college/AA degree	1.27	1.23	1.25	1.27	1.23
	(1.00, 1.61)	(0.97, 1.56)	(0.99, 1.59)	(0.99, 1.61)	(0.97, 1.56)
Race/Ethnicity (REF: Any Other Race)					
Non-Hispanic White	1.75 ***	1.73 ***	1.79 ***	1.75 ***	1.76 ***
	(1.39, 2.21)	(1.37, 2.19)	(1.37, 2.19)	(1.36, 2.16)	(1.40, 2.21)
Metropolitan Residence (REF: Metropolitan)					
Non-metropolitan	2.09 ***	1.92 ***	1.92 ***	2.09 ***	1.90 ***
	(1.62, 2.70)	(1.48, 2.48)	(1.42, 2.26)	(1.62, 2.69)	(1.47, 2.46)
Sensation Seeking	1.41 ***	1.43 ***	1.43 ***	1.41 ***	1.43 ***
	(1.24, 1.60)	(1.26, 1.63)	(1.25, 1.62)	(1.24, 1.60)	(1.26, 1.63)
Own Poly-Tobacco Use (REF: None)					
Any	3.00 ***	2.98 ***	2.99 ***	3.00 ***	2.98 ***
	(2.47, 3.65)	(2.46, 3.63)	(2.46, 3.64)	(2.47, 3.64)	(2.46, 3.63)
Household Tobacco Use (REF: None)					
Any	1.27 **	1.27 **	1.27 **	1.27 **	1.27 **
	(1.08, 1.50)	(1.08, 1.50)	(1.08, 1.50)	(1.08, 1.50)	(1.08, 1.50)
Peer Smoking (REF: None)					
1	1.70 ***	1.67 ***	1.69 ***	1.69 ***	1.67 ***
	(1.38, 2.08)	(1.36, 2.06)	(1.37, 2.08)	(1.38, 2.08)	(1.36, 2.06)
2	2.49 ***	2.41 ***	2.47 ***	2.48 ***	2.41 ***
	(1.97, 3.15)	(1.91, 3.05)	(1.95, 3.12)	(1.96, 3.14)	(1.91, 3.05)
3	2.36 ***	2.29 ***	2.33 ***	2.36 ***	2.29 ***
	(1.70, 3.29)	(1.64, 3.20)	(1.67, 3.24)	(1.69, 3.28)	(1.64, 3.19)
4	1.97 **	1.84 **	1.94 **	1.96 **	1.84 **
	(1.34, 2.88)	(1.26, 2.69)	(1.32, 2.84)	(1.33, 2.87)	(1.26, 2.69)
Real Weighted Avg Price of Smokeless Tobacco (USD per one ounce)		0.79 ***			0.79 ***
		(0.73, 0.85)			(0.72, 0.87)
Smoke-Free Indoor Air Laws (% population)			0.44 **		0.85
			(0.28, 0.70)		(0.50, 1.45)
Real State Tobacco Control Expenditure (USD per capita)				0.96	1.02
				(0.81, 1.10)	(0.97, 1.08)
Wave					
8	0.94	0.98	0.94	0.94	0.98
	(0.81, 1.10)	(0.84, 1.14)	(0.81, 1.10)	(0.81, 1.10)	(0.84, 1.15)
9	0.98	1.04	0.98	0.97	1.04
	(0.82, 1.16)	(0.87, 1.24)	(0.82, 1.16)	(0.82, 1.15)	(0.87, 1.24)
State Fixed Effects Included	No	No	No	No	No
Model Number of Observations	34,201	34,121	34,201	34,201	34,121
Model Number of Survey Participants	15,089	15,056	15,089	15,089	15,056
Model Mean VIF	1.20	1.20	1.20	1.20	1.22

Notes: * *p* < 0.05, ** *p* < 0.01, *** *p* < 0.001, 95% confidence intervals are shown in parenthesis. A GEE logistic regression modeling technique was used.

## Data Availability

Data cannot be shared publicly due to IRB restrictions. Data are available from the Truth Initiative for researchers who meet the criteria for access to confidential data.

## Supplementary Materials

The following supporting information can be downloaded at: https://www.mdpi.com/article/10.3390/ijerph19084786/s1, Table S1: Current smokeless tobacco use modeled against tobacco advertising expenditure, social media use, sociodemographic characteristics and policy variables with state and wave fixed effects using Waves 7, 8 and 9 (2017–2019) of the national Truth Longitudinal Cohort Study.
