# Supplementary material for: Online Tobacco Advertising and Current Chew, Dip, Snuff and Snus Use among Youth and Young Adults, 2018–2019
Source: Int J Environ Res Public Health. 2022 Apr 14;19(8):4786. doi: 10.3390/ijerph19084786 (PMC9026367; doi:10.3390/ijerph19084786)
Supplement: Supplementary file 1 [file ijerph-19-04786-s001.zip › ijerph-1619970-supplementary.pdf]

International Journal of
Environmental Research and Public Health

MDPI

Supplementary Table S1. Current smokeless tobacco use modeled against tobacco advertising expenditure, social media use, sociodemographic characteristics and policy variables with state and wave fixed effects using Waves 7, 8 and 9 (2017–2019) of the national Truth Longitudinal Cohort Study.

	**Model 6**	**Model 7**	**Model 8**	**Model 9**
	**OR**	**OR**	**OR**	**OR**
	**95% CI**	**95% CI**	**95% CI**	**95% CI**
Tobacco Ad Exposure (REF: Never)				
Sometimes	1.18 ** (1.01, 1.37)	1.17 ** (1.01, 1.36)	1.17 * (1.00, 1.38)	1.03 (0.87, 1.23)
Often, Very often	2.47 *** (1.98, 3.09)	2.45 *** (1.96, 3.05)	2.51 *** (1.97, 3.19)	2.04 *** (1.61, 2.59)
Social Media Use (REF: 3 or More Hours)				
None		0.58 *** (0.39, 0.87)	0.52 *** (0.35, 0.78)	0.65 ** (0.43, 0.98)
Less than 1 h		0.98 (0.80, 1.19)	0.84 (0.67, 1.04)	0.94 (0.76, 1.18)
1 to 3 h		0.98 (0.83, 1.16)	0.91 (0.76, 1.10)	0.97 (0.80, 1.18)
Age			1.03 ** (1.00, 1.06)	1.02 (1.00, 1.05)
Gender (REF: Female)				
Male			6.03 *** (4.70, 7.75)	5.56 *** (4.305, 7.18)
Parent Education (REF: College Graduate or More)				
Less than high school			1.53 * (0.93, 2.52)	1.32 (0.79, 2.23)
High school graduate			1.46 ** (1.08, 1.99)	1.11 (0.81, 1.54)
Some college/AA degree			1.54 *** (1.22, 1.93)	1.22 (0.96, 1.55)
Race/Ethnicity (REF: Any Other Race)				
Non-Hispanic White			1.75 *** (1.38, 2.21)	1.66 *** (1.30, 2.12)
Metropolitan Residence (REF: Metropolitan)				
Non-metropolitan			2.16 *** (1.69, 2.76)	1.74 *** (1.33, 2.28)
Sensation Seeking			1.77 *** (1.56, 2.00)	1.44 *** (1.26, 1.64)
Household Tobacco Use (REF: None)				
Any				1.27 *** (1.08, 1.50)
Peer Smoking (REF: None)				
1				1.67 *** (1.34, 2.05)
2				2.39 ***

				(1.89, 3.03)
3				2.29 *** (1.63, 3.20)
4				1.82 *** (1.24, 2.67)
Own Poly-Tobacco Use (REF: None)				
Any				3.03 *** (2.49, 3.68)
Real Weighted Average Price of Smokeless Tobacco (USD per one ounce)				0.80 (0.40, 1.63)
Smoke-Free Indoor Air Laws (% population)				1.76 (0.00, 4061.92)
Real State Tobacco Control Expenditure (USD per capita)				1.02 (0.86, 1.20)
Wave				
8	0.97 (0.86, 1.10)	0.97 (0.86, 1.10)	0.91 (0.79, 1.05)	0.98 (0.80, 1.21)
9	0.98 (0.86, 1.12)	0.98 (0.86, 1.12)	0.91 (0.78, 1.07)	1.03 (0.78, 1.37)
State Fixed Effects Included	No	No	No	Yes
Model Number of Observations	37,132	37,052	34,505	33,716
Number of Survey Participants	15,934	15,923	15,131	14,895
Model Mean VIF	1.14	1.31	1.21	46.91

Notes: * $p < 0.05$, ** $p < 0.01$, *** $p < 0.001$, 95% confidence intervals are shown in parenthesis.
